# Inhibition of tooth demineralization caused by *Streptococcus mutans* biofilm via antimicrobial treatment using hydrogen peroxide photolysis

**DOI:** 10.1007/s00784-022-04821-2

**Published:** 2022-12-09

**Authors:** Midori Shirato, Keisuke Nakamura, Taichi Tenkumo, Yoshimi Niwano, Taro Kanno, Keiichi Sasaki, Peter Lingström, Ulf Örtengren

**Affiliations:** 1 Department of Cariology, Institute of Odontology, Sahlgrenska Academy, University of Gothenburg, 405 30 Gothenburg, Sweden; 2 Department of Advanced Free Radical Science, Tohoku University Graduate School of Dentistry, 4-1 Seiryo-Machi, Aoba-Ku, Sendai, 980-8575 Japan; 3 Division of Advanced Prosthetic Dentistry, Tohoku University Graduate School of Dentistry, 4-1 Seiryo, Aoba-Ku, Sendai, 980-8575 Japan; 4 Faculty of Nursing, Shumei University, 1-1 Daigaku-Cho, Yachiyo, 276-0003 Japan

**Keywords:** Biofilms, Demineralization, Dental caries, Hydrogen peroxide, *Streptococcus mutans*

## Abstract

**Objectives:**

An antimicrobial technique utilizing hydroxyl radicals generated by the photolysis of 3% H_2_O_2_ has been developed recently. The present study aimed to evaluate the effect of H_2_O_2_ photolysis treatment on tooth demineralization caused by *Streptococcus mutans* biofilm.

**Materials and methods:**

To induce tooth demineralization, *S. mutans* biofilm was allowed to form on the maxillary first molars collected from Wistar rats via 24-h culturing. The samples were immersed in 3% H_2_O_2_ and irradiated with 365-nm LED (H_2_O_2_ photolysis treatment). Viable bacterial counts in the biofilm were evaluated immediately after treatment and after an additional 30-h culturing by colony counting. The acidogenicity of the biofilm, re-established 30 h after treatment, was assessed by measuring the pH. The effect of H_2_O_2_ photolysis treatment on tooth demineralization was assessed by measuring the depth of the radiolucent layer in micro-CT images.

**Results:**

H_2_O_2_ photolysis significantly reduced viable bacterial counts in the biofilm to 3.7 log colony forming units (CFU)/sample, while the untreated group had 7.9 log CFU/sample. The pH of the biofilm re-established after treatment (6.6) was higher than that of the untreated group (5.3). In line with the pH measurement, the treatment group had a significantly lower depth of radiolucent layer in dentin than the untreated group.

**Conclusions:**

H_2_O_2_ photolysis treatment was effective not only in killing the biofilm-forming *S. mutans* but also in lowering the acidogenicity of the biofilm. Thus, this technique could inhibit tooth demineralization.

**Clinical relevance:**

H_2_O_2_ photolysis can be applicable as a new dental caries treatment.

## Introduction

Dental caries is a tooth demineralization caused by organic acids produced by acidogenic bacteria, such as *Streptococcus mutans*, present in the dental plaque [1–3]. Although the prevalence of dental caries has declined in the younger generation [4], it still persists as a common and major health issue worldwide [5, 6]. Dental plaque plays a key role in the development of dental caries in conjunction with other factors such as dietary sugar consumption and buffering capacity of saliva. Hence, effective elimination and/or inactivation of dental plaque, a microbial biofilm, is crucial for the prevention and treatment of dental caries. Cariogenic bacteria in dental plaque demineralize the enamel and invade dentin, resulting in the formation of infected and affected dentin [7, 8]. In general, microbial biofilms and infected dentin can be mechanically removed by instrumentation. However, in some cases, adequate removal is difficult by mechanical means alone due to the complicated anatomical configuration of teeth and difficulty in distinguishing between healthy and infected dentin [9]. Accordingly, adjunctive antimicrobial chemotherapy that inactivates the microbial biofilms is desirable [10].

An antimicrobial technique utilizing the photochemical reaction of hydrogen peroxide (H_2_O_2_) has been developed [11–13]. This technique utilizes the bactericidal action of hydroxyl radicals generated by the photolysis of 3% H_2_O_2_ [11, 12]. In the medical and dental fields, 3% H_2_O_2_ is a widely used disinfectant for skin and oral mucosa [14]. Although the antimicrobial activity of 3% H_2_O_2_ is not sufficient to treat dental infectious diseases, hydroxyl radicals generated via 3% H_2_O_2_ photolysis can be used effectively because of their potent bactericidal activity [11, 15]. In the process of H_2_O_2_ photolysis, the O–O covalent bond in H_2_O_2_ is homolytically cleaved by photo-irradiation at a wavelength of ≤ 405 nm, resulting in the generation of hydroxyl radicals. Hydroxyl radicals are potent oxidants with a redox potential of 2.80 V, which is higher than that of ozone (2.08 V), and H_2_O_2_ (1.78 V) [16]. When these hydroxyl radicals react with microorganisms, they cause lethal damage to the bacteria via non-selective oxidation of cell components such as lipid membrane, DNA, and protein [17, 18]. The bactericidal effect of H_2_O_2_ photolysis has been proven effective against various oral microbes including *S. mutans* [11, 12].

Concerning the safety aspect of the technique, it was reported that no inflammatory cell infiltration was observed when the oral mucosa of rats and hamsters were treated with photolysis of 3% H_2_O_2_ using 405-nm laser at a power of < 80 mW, thus proving it safe [19–21]. Besides, when a rat tooth was treated with photolysis of 3% H_2_O_2_ using 365-nm LED at an irradiance of 2000 mW/cm^2^, infiltration of inflammatory cells in the dental pulp was not observed either [22]. Thus, it is considered that the acute, locally injurious properties of H_2_O_2_ photolysis against dental tissues may be clinically acceptable. In addition, the residual toxicity may be negligible because of the use of lower concentration of H_2_O_2_ (3%) [14] and the short lifetime of hydroxyl radicals [23]. Furthermore, a literature review concluded that there is almost no risk of carcinogenicity as long as the hydroxyl radicals are used as antimicrobials in the oral cavity for a short duration of time [24]. Based on these findings, a clinical trial was conducted, which demonstrated that the adjunctive use of H_2_O_2_ photolysis treatment was effective in the nonsurgical treatment of moderate to severe periodontitis [25]. Thus, it is contemplated that this technique can also be used for prophylaxis and/or treatment of dental caries.

H_2_O_2_ photolysis exerts a bactericidal effect against the cariogenic biofilm. Nakamura et al. demonstrated that treatment for 1 min with 3% H_2_O_2_ photolysis using ultraviolet light with a wavelength of 365 nm resulted in over 5 log reduction in the viable bacterial counts of biofilm-forming *S. mutans*, despite the biofilm acquiring antibiotic resistance [26]. Shirato et al. investigated the time-kill kinetics of H_2_O_2_ photolysis against *S. mutans* biofilm compared to conventional antiseptics used in the oral cavity, such as 0.2% chlorhexidine gluconate and 0.5% povidone-iodine [15]. It was demonstrated that the decimal reduction value of 3% H_2_O_2_ photolysis using 365-nm light-emitting diode (LED) was 0.06 min, whereas that of chlorhexidine gluconate and povidone-iodine were 10.19 and 6.40 min, respectively, suggesting that the former was much more effective in killing biofilm-forming *S. mutans*. However, it is still unclear whether the antimicrobial treatment using H_2_O_2_ photolysis can prevent or inhibit the development of dental caries.

Tooth demineralization can be detected using radiographic procedures as there is a difference between the X-ray absorption rates of intact and demineralized teeth [27]. Micro-computed tomography (CT) is a powerful tool providing high resolution and three-dimensional information of dental caries in rodents [27–29]. Therefore, the present study aimed to evaluate the inhibitory effect of H_2_O_2_ photolysis on demineralization in rat teeth caused by *S. mutans* biofilm using micro-CT technique. The hypothesis was that H_2_O_2_ photolysis would reduce the number of viable *S. mutans* in the biofilm formed on the tooth surface, and thus, would prevent or deter the demineralization process caused by the bacterial activity.

## Materials and methods

### Sample preparation

**Fig. 1:**
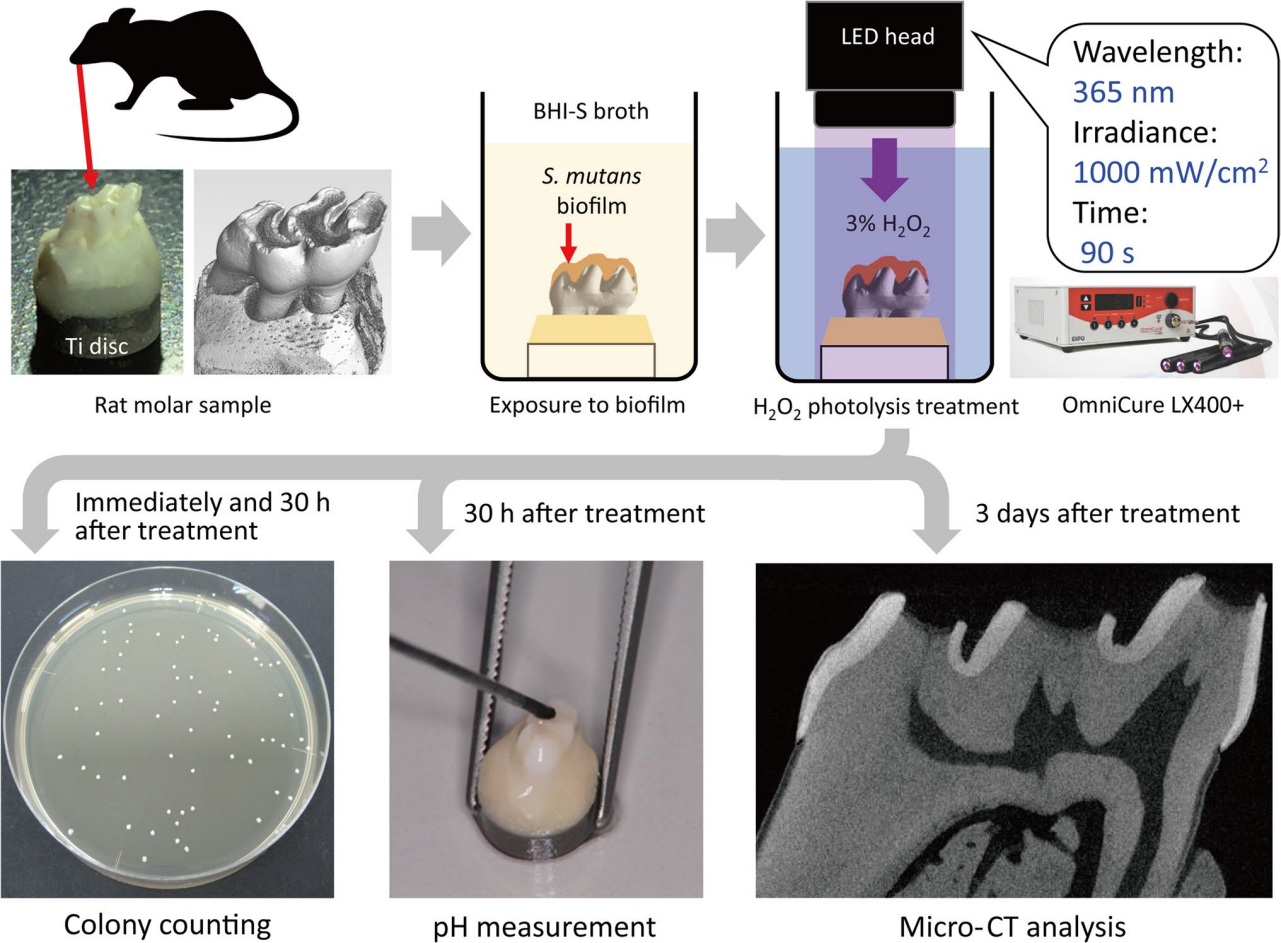
Study design. Rat maxillary first molars were fixed on titanium discs using resin. The molar sample was immersed in a suspension of *Streptococcus mutan*s in brain heart infusion broth supplemented with 1% sucrose (BHI-S) to form a biofilm on the tooth surface. The sample containing the biofilm was immersed in 3% hydrogen peroxide and irradiated with 365-nm LED for 90 s (H_2_O_2_ photolysis treatment). Viable bacterial counts in the biofilm were evaluated by colony counting immediately after the treatment and after an additional 30 h of culturing. The acidogenicity of the biofilm re-established 30 h after treatment was assessed by pH measurement using a microelectrode. The effect of H_2_O_2_ photolysis treatment on tooth demineralization caused by the *S. mutans* biofilm was analyzed using micro-CT

To obtain intact teeth, maxillary first molars with surrounding bone were collected from male Wistar rats (10–14-week-old) participating in other animal studies without interventions to the teeth or oral cavity. The protocols for the animal experiments were reviewed and approved by the Institutional Animal Experiment Committee of Tohoku University (approval number: 2017DnA-047). All experimental procedures were conducted in accordance with the guidelines for animal experiments adopted by Tohoku University. The molar, with surrounding tissue, was dissected and fixed on a titanium disk using self-curing acrylic resin (UNIFAST III, GC, Tokyo, Japan) so that the samples would not float in the liquid medium (Fig. 1). The samples were then immersed in saline and autoclaved at 121 °C for 15 min (LSX-300, Tomy Seiko, Tokyo, Japan).

To induce initial caries-like demineralization (hereafter referred to as initial caries), *S. mutans* biofilms were allowed to form on the molars, in accordance with a previous study [15]. Briefly, the test strain, *S. mutans* JCM 5705, obtained from the Japan Collection of Microorganisms (RIKEN BioResource Center, Wako, Japan), was incubated anaerobically using AneroPack (Mitsubishi Gas Chemical Company, Tokyo, Japan) in brain heart infusion (BHI) broth (Becton Dickinson, Franklin Lakes, NJ, USA) at 37 °C for 24 h. Bacteria pre-incubated in the broth were harvested by centrifugation at 1400 × *g* for 5 min and re-suspended in sterile saline to obtain 10^8^ colony-forming units (CFU)/mL. The bacterial suspension (100 µL) was added to 1000 µL of BHI broth supplemented with 1% sucrose (BHI-S) in each well of a 48-well plate (i.e., initial bacterial count = 10^7^ CFU). Subsequently, an autoclaved molar sample was immersed in each well and the 48-well plate with the samples was anaerobically incubated at 37 °C for 24 h, to allow formation of biofilms on the samples. The samples were then used for assays.

The present study mainly comprised of two parts: (i) characterization of the in vitro caries model; (ii) evaluation of the effect of H_2_O_2_ photolysis treatment on tooth demineralization using the caries model. The study design for the second part is illustrated in Fig. 1.

### Characterization of rat molars demineralized by S. mutans biofilm

#### Micro-CT analysis

**Fig. 2:**
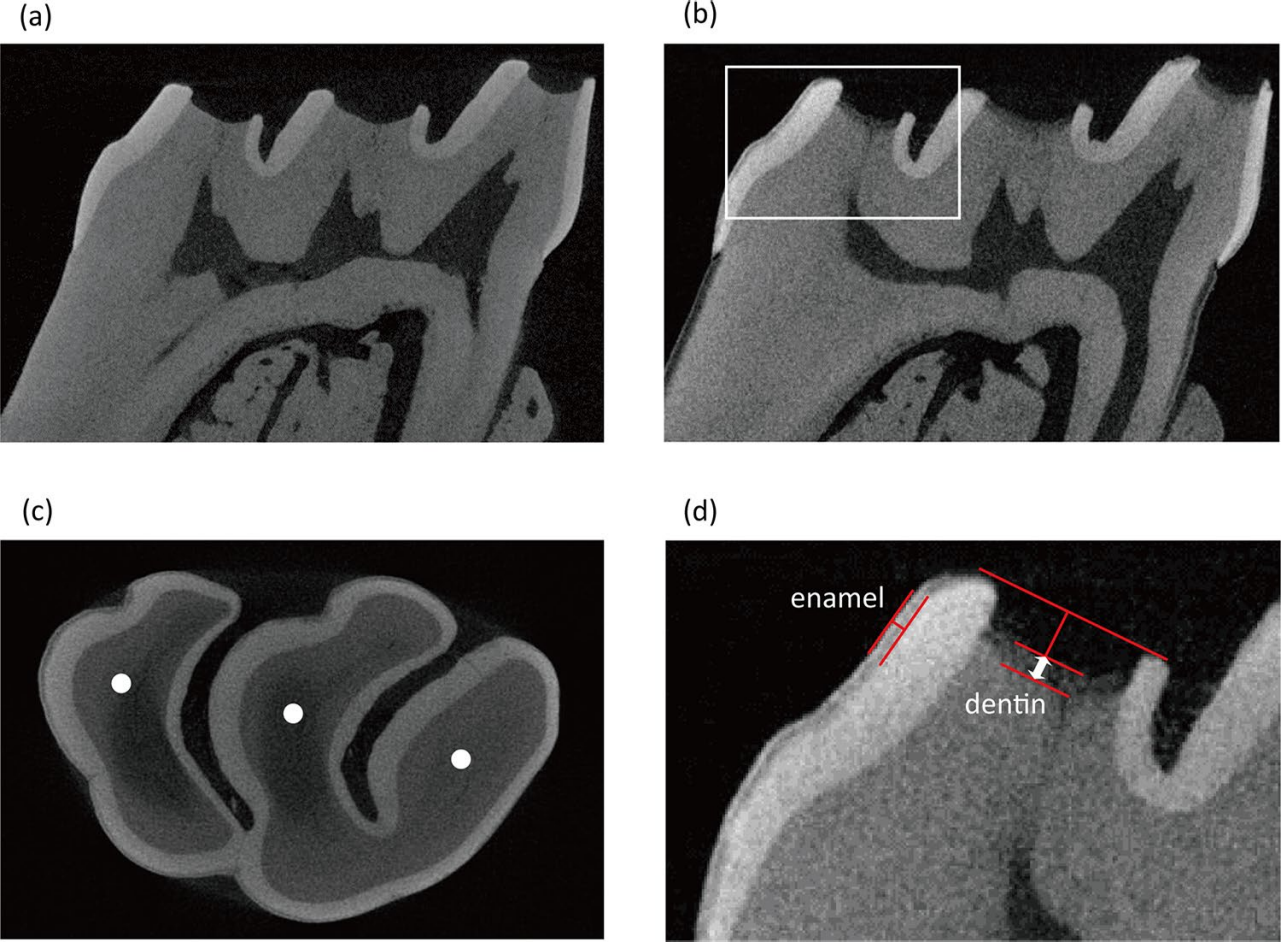
Measurement of the depth of the radiolucent layer produced by *Streptococcus mutans* biofilm. Representative micro-computed tomography images of rat molar samples obtained before (**a**) and after 24-h exposure to *S. mutans* biofilm in brain heart infusion broth supplemented with 1% sucrose (**b**). The depth of the radiolucent layer was measured at the central part of each cusp, as shown by the white circles (**c**). The area under the white square in (**b**) is shown at a higher magnification in (**d**). The depth was determined as the distance between the surface and bottom of the radiolucent layer. A reference line connecting the edges of the enamel layer in each cusp was drawn when the top of the dentin surface was unclear due to demineralization. The depth of the affected dentin was determined by subtracting the distance between the reference line and the top of the dentin surface in the image obtained before exposure to the *S. mutans* biofilm from the distance between the reference line and the bottom of the radiolucent layer in the image obtained after exposure to the *S. mutans* biofilm

Six samples were analyzed using micro-CT before and after the formation of the *S. mutans* biofilm (ScanXmate-D225RSS270, Comscantecno, Japan). The samples with *S. mutans* biofilm were autoclaved at 121 °C for 15 min before micro-CT analysis, to annul the demineralization effects of the biofilm. The measurement conditions were as follows: voltage, 120 kV; current, 80 µA; resolution, 6.0 µm/voxel; projection, 1000; and scan speed, 6 flames/sec. The CT data were reconstructed using a device software (Cone CT Express, Comscantecno). The black and white values were set as −70 and 400, respectively. No filters were used for reconstruction to alleviate an artifact. The progression of demineralization (i.e., initial caries) was evaluated as the depth of the radiolucent layer in enamel and dentin, which was measured at the central part of each cusp using an image-processing program (ImageJ; Research Services Branch of the National Institutes of Health, Bethesda, MD, USA). Depth of the radiolucent layer in enamel was determined as the distance between the surface and bottom of the layer (Fig. 2). For measurement in dentin, a reference line connecting the edges of the enamel layer in each cusp was drawn. Depth of the affected dentin was determined using the reference line, as shown in Fig. 2. The measurements were performed by two trained examiners (MS and KN), and the coefficient of variation (CV) for the measurement was evaluated. The CV was approximately 5% for both examiners, indicating that the accuracy of measurement was acceptable.

In addition, the mineral density of the radiolucent layer and region was analyzed. For this analysis, a reference phantom containing different concentrations of hydroxyapatite in the range of 200–1600 mg/cm^3^ (Ratoc System Engineering, Tokyo, Japan) was additionally scanned. Based on the image obtained for the phantom, a standard curve of gray values was constructed as a function of mineral density. Then, the mineral density of the radiolucent layer and intact region was calculated.

#### Element analysis

**Fig. 3:**
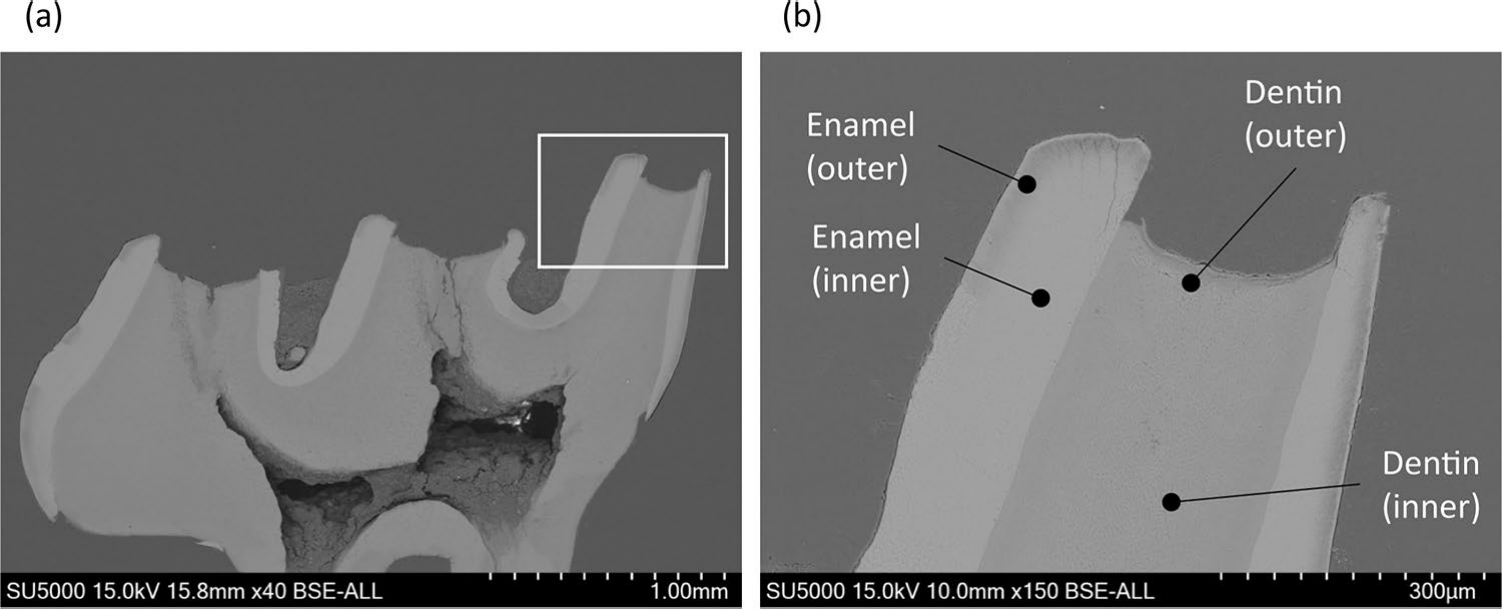
Representative scanning electron microscope (SEM) images showing analytical points for energy-dispersive X-ray spectroscopy (EDS). The area under the white square in **a** is shown at a higher magnification in **b**. The outer regions of enamel and dentin were defined as the “affected regions,” while the inner regions were defined as “intact regions”

Eight samples were prepared as described above and autoclaved at 121 °C for 15 min after an incubation period of 24 h. The samples were embedded in epoxy resin (Sankei, Tokyo, Japan). Cross sections were prepared in the mesiodistal direction, coated with gold, and analyzed using a scanning electron microscope (SEM) (SU5000, Hitachi, Japan) with a detector for energy-dispersive X-ray spectroscopy (EDS) (EDAX Pegasus EDS/EBSP, Ametek, Berwyn, PA, USA). The samples were observed using SEM operated at 15 kV. The outer regions of enamel and dentin were defined as the “affected region,” while the inner regions were referred to as the “intact region” (Fig. 3). EDS spectra of each region were obtained at a magnification of 800 × with a resolution of 126 eV for element analysis of the “intact” and “affected” regions of enamel and dentin.

### Effect of H_2_O_2_ photolysis on S. mutans biofilm

#### Viable bacterial counts in S. mutans biofilms treated with H_2_O_2_ photolysis

The biofilm samples prepared as described above were divided into four groups (*n* = 12 for each group), which comprised of single or combined treatments using LED irradiation (*L*) and immersion in 3% H_2_O_2_ (*H*). Thus, the test groups were denoted L( +)H( +), L( +)H( −), L( −)H( +), and L( −)H( −). L( +) samples were irradiated with 365 nm LED at an irradiance of 1000 mW/cm^2^ using an LED spot-curing device (OmniCure LX400 + , Lumen Dynamics Group, Mississauga, ON, Canada), whereas L( −) samples were kept in a light-shielding box. H( +) samples were immersed in 500 µL of 3% H_2_O_2_ prepared by diluting 30% H_2_O_2_ (Santoku Chemical, Tokyo, Japan) with pure water obtained from a water purification system (Synergy UV, Millipore, Darmstadt, Germany), whereas H( −) samples were immersed in 500 µL of pure water. Thus, L( −)H( −) was used as the control group. Unless otherwise mentioned, L( +)H( +) and L( +)H( −) treatments were performed using 365 nm LED, and all treatments were performed for 90 s. After treatment, the samples were washed twice with saline to eliminate the effect of H_2_O_2_. Half of the samples in each group (*n* = 6) were subjected to determination of viable bacterial counts immediately after treatment, while the remaining samples were additionally cultured in BHI-S for 30 h under the same conditions as described above, followed by the determination of viable bacterial counts. For viable counts, the biofilms were detached from the rat molar using an enzymatic technique according to previous studies [30, 31]. The samples were immersed in 500 μL of an enzyme solution, composed of 4 mg/mL type I collagenase (Thermo Fisher Scientific, Waltham, MA, USA) and 2 mg/mL dispase (Thermo Fisher Scientific) in phosphate-buffered saline. The 48-well plate containing the samples was incubated for 2 h under rotation at 200 rpm at 37 °C. After the incubation period, the sample and enzyme solution were transferred to a 1.5-mL microtube, and the tube was vortexed at 2500 rpm for 10 s. The mixture was serially diluted 10-folds in saline, and 10 μL of the dilution was inoculated onto BHI agar. The agar plates were cultured anaerobically at 37 °C for 24 h, and colony counting was performed to determine the CFU/sample.

#### Acidogenicity of S. mutans biofilm treated with H_2_O_2_ photolysis

Twelve samples were prepared and divided into four treatment groups (*n* = 3 for each group). Treatment was carried out using the same method as described earlier and the treated samples were cultured in BHI-S for additional 30 h. After 30-h incubation, the samples were transferred to fresh BHI-S and incubated for another 30 min, and the pH of the biofilm was measured using a pH meter (Seven Go pH meter SG2, Mettler Toledo) with a microelectrode (650 µm in diameter; AMANI-650, Innovative Instruments, Tampa, FL, USA). To measure the pH value of the inner biofilms, BHI-S was removed, and the tip of the microelectrode was directly inserted into the biofilms.

### Effect of H_2_O_2_ photolysis on tooth demineralization caused by S. mutans biofilm

#### Inhibitory effect of H_2_O_2_ photolysis on demineralization

Thirty samples were prepared and divided into five groups, comprising a baseline group and four treatment groups. The baseline group (initial caries group) was subjected to autoclaving (121 °C for 15 min) without any treatment and additional incubation. The remaining four groups were treated using the same method as described earlier. Then, the samples were washed twice with saline to eliminate the effect of H_2_O_2_ and immersed in a fresh medium (1000 µL of BHI-S), followed by an additional 3-day incubation to allow further demineralization. During this period, the BHI-S was replaced with a fresh solution every day. After the incubation period, all samples were autoclaved to eliminate the effect of *S. mutans* biofilm. Demineralization was analyzed using micro-CT, and the depth of the radiolucent layer was measured by two calibrated examiners, as described earlier. The concordance rate of the measurements within an error range of 20 µm (approximately 3 pixels in a micro-CT image) was relatively high (91% for enamel and 72% for dentin), and the mean value of the measurements of both the examiners was regarded as the representative value for each sample.

#### Influence of irradiation time and wavelength of light

The yield of hydroxyl radicals generated by H_2_O_2_ photolysis are dependent on the irradiation time and wavelength of light [32]; thus, their influence on demineralization was additionally evaluated. Photoirradiation was performed for 30 s or 90 s using LED heads that emitted light at wavelengths of 365 nm and 400 nm. Thus, the treatment groups examined in this assay were treated for 30 s with L( +)H( +) at 365 nm, 90 s with L( +)H( +) at 365 nm, and 90 s with L( +)H( +) at 400 nm. In addition, a group that underwent 90-s treatment with L( −)H( −) was included as control. The treatments and micro-CT analyses were performed using the same methods as described earlier.

### Sample size calculation

Sample size was calculated to determine the number of samples used for each analysis using JMP Pro 16.0 (SAS Institute, Cary, NC). The type I error (α) and type II error (β) were set at 0.05 and 0.2 (i.e., power = 0.8), respectively. We assumed mean values and standard deviations in the sample size calculations for each analysis based on our preliminary study findings. According to the calculations, the total sample sizes were determined to be 8 for element analysis (*n* = 8), 24 for viable bacterial counts (*n* = 6), 12 for pH measurement (*n* = 3), and 30 for micro-CT analysis (*n* = 6).

### Statistical analyses

Statistical analyses were performed using JMP Pro 16 (SAS Institute, Cary, NC, USA). The CFU data were converted to logarithmic values for statistical analysis, while other numerical data were used without conversion. First, the distribution of data and homogeneity of variance were examined using the Shapiro–Wilk and Levene’s tests, respectively. These tests revealed that the data were normally distributed; however, the variances in some comparison groups were not homogeneous. Thus, differences were assessed using Welch’s *t*-test for pairwise comparisons or the Games–Howell test for multiple comparisons, both of which can be used to compare the differences between the groups even when the assumption of homogeneity of variances is violated. Statistical significance was set at *p* < 0.05.

## Results

### Characterization of rat molars demineralized by S. mutans biofilm

**Fig. 4:**
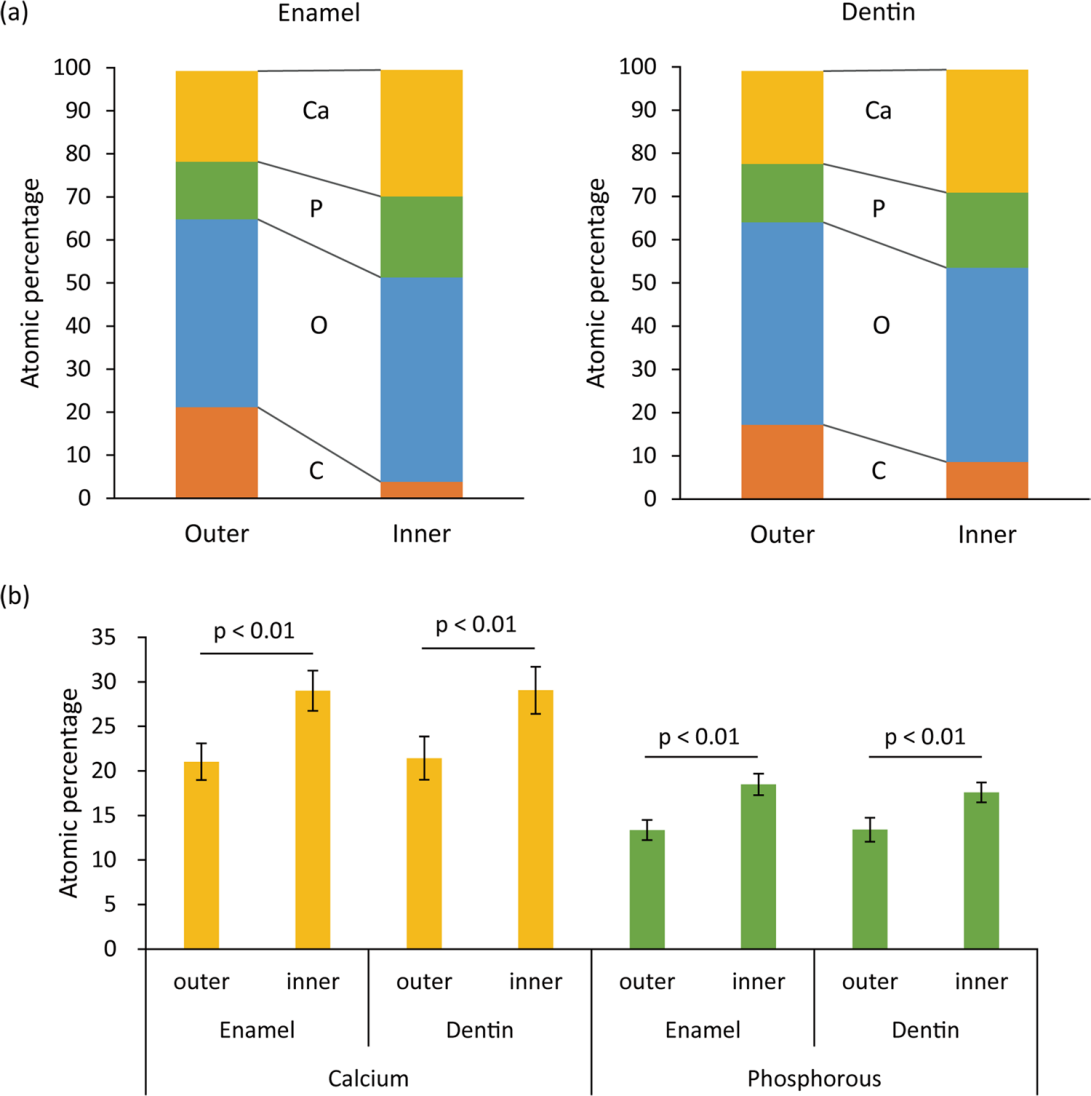
Elemental composition of rat molars. Elemental analysis was performed using a scanning electron microscope with a detector for energy-dispersive X-ray spectroscopy. Calcium (Ca), phosphorous (P), oxygen (O), and carbon (C) were mainly detected in the enamel and dentin of rat molars (**a**). The “affected” (outer) region was characterized by lower amounts of Ca and P and a higher amount of C than the “intact” (inner) region. The atomic percentages of Ca and P in the outer region of both enamel and dentin were significantly lower than the inner region (**b**). The values and error bars indicate the mean and standard deviation, respectively (*n* = 8 for each group)

Micro-CT analysis revealed that 24 h incubation with *S. mutans* resulted in the generation of a radiolucent layer on the outer surface of both enamel and dentin. The average depth of the radiolucent layer in enamel and dentin were 24.5 and 74.3 µm, respectively. The mineral density of the intact enamel was 1620.2 mg/cm^3^ (SD: 483.2). In contrast, the mineral density of the radiolucent layer was 932.9 mg/cm^3^ (SD: 318.2), suggesting demineralization. Analogously, mineral densities in the radiolucent layer and intact region of dentin were 317.4 mg/cm^3^ (SD: 142.3) and 739.0 mg/cm^3^ (SD: 252.4), respectively. The differences in mineral density between the intact region and radiolucent layer detected in enamel and dentin were statistically significant (*p* < 0.05). According to the EDS analysis, the elemental composition of the intact and affected regions were different (Fig. 4a). The affected region corresponding to the radiolucent layer in micro-CT analysis showed a significantly lower percentage of calcium and phosphorous as compared to the intact region (Fig. 4b).

### Effect of H_2_O_2_ photolysis on S. mutans biofilm and acidogenicity of the biofilm

**Fig. 5:**
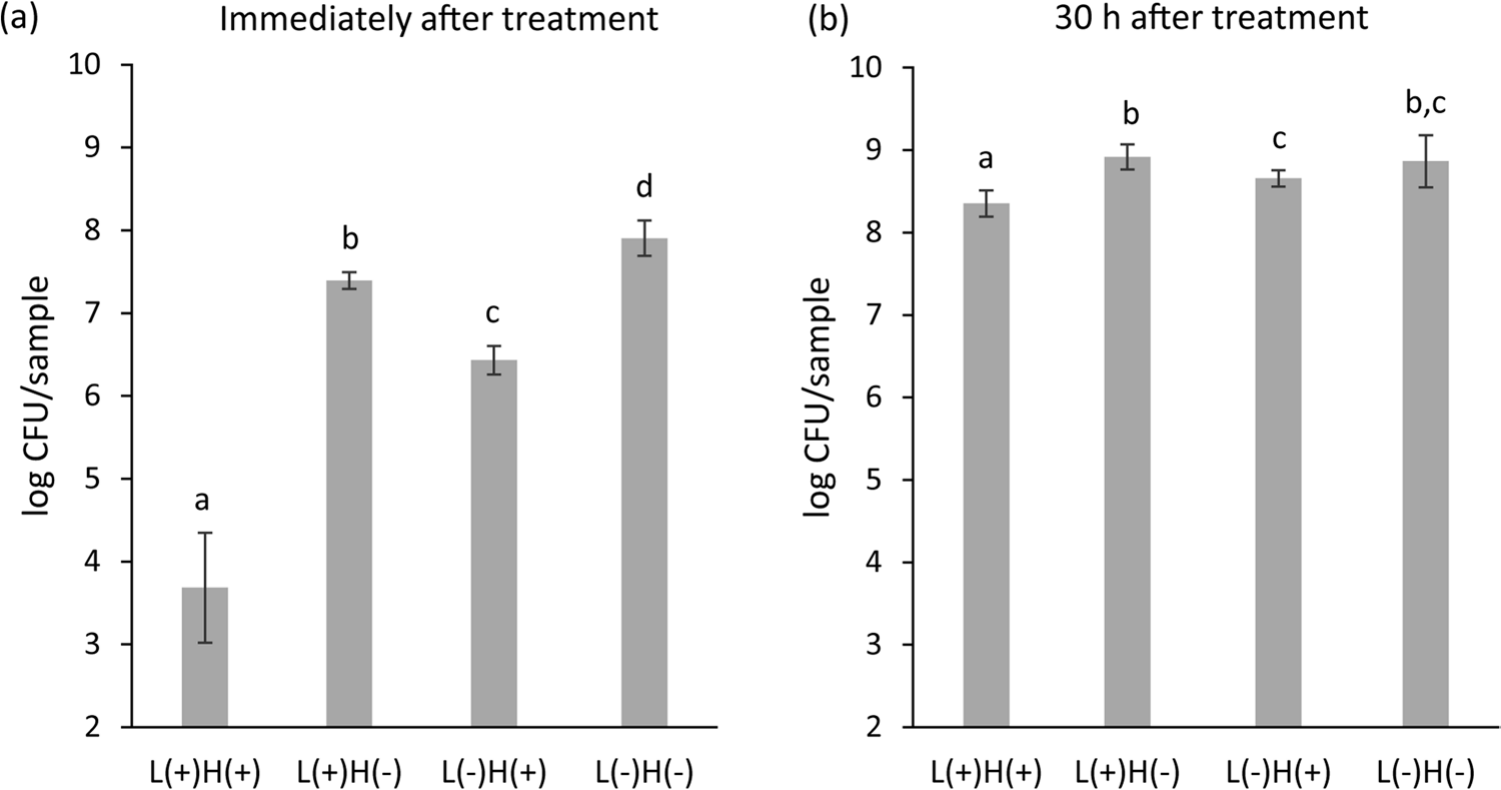
Viable bacterial counts in *Streptococcus mutans* biofilm immediately and 30 h after treatment. When the biofilm formed on the rat molar was subjected to 90 s treatment of 365-nm LED irradiation of 3% H_2_O_2_ [L( +)H( +)], viable bacterial counts evaluated immediately after treatment were significantly lower than the other treatment groups (**a**). After an additional 30 h of culturing, the viable bacterial counts in the biofilm reached over 8 log CFU/sample, regardless of the treatment groups (**b**). The values and error bars indicate the mean and standard deviation, respectively (*n* = 6 for each group). Different letters above the columns indicate significant differences (*p* < 0.05) between the different groups

Within 90 s of treatment, L( +)H( +) significantly reduced the number of viable *S. mutans* biofilms by over 4 log CFU/sample, as compared to L( −)H( −) (Fig. 5a). In addition, L( +)H( −) and L( −)H( +) significantly decreased the viable bacterial counts; however, the reduction was less than 1.5 log CFU/sample. After additional culturing of 30 h following the 90 s treatment, surviving bacterial re-growth and biofilm re-establishment were examined. The viable counts in the re-established biofilms treated with L( +)H( +), L( +)H( −), L( −)H( +), and L( −)H( −) were 8.4, 8.7, 8.9, and 8.9 log CFU/sample, respectively (Fig. 5b). Although the difference was small, the viable counts for the L( +)H( +) group were significantly lower than the other groups.

**Fig. 6:**
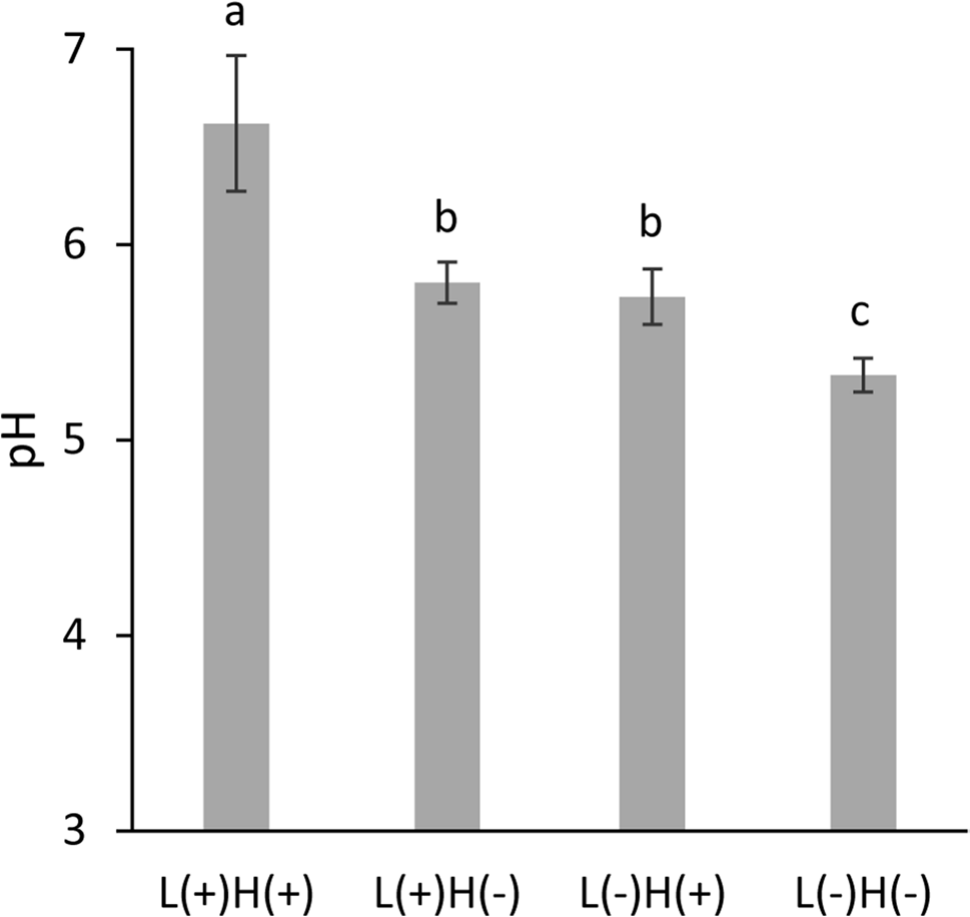
Measurement of pH in *Streptococcus mutans* biofilm re-established 30 h after treatment. The biofilm re-established after 90 s treatment with 365-nm LED irradiation of 3% H_2_O_2_ [L( +)H( +)] showed a significantly higher pH (6.6) compared to the other treatment groups (i.e., the lowest acidogenicity). The values and error bars indicate the mean and standard deviation, respectively (*n* = 3 for each group). Different letters above the columns indicate significant differences (*p* < 0.05) between different groups

The acidogenicity of the biofilms varied depending on the treatment group. When exposed to fresh BHI-S (pH 7.4) for 30 min, the pH of the re-established biofilms after treatment with L( +)H( +), L( +)H( −), L( −)H( +), and L( −)H( −) were 6.6, 5.8, 5.7, and 5.3, respectively (Fig. 6). Thus, the acidogenicity of the biofilm treated with L( +)H( +) was significantly lower than biofilms exposed to other treatments.

### Effect of H_2_O_2_ photolysis on tooth demineralization caused by S. mutans biofilm

**Fig. 7:**
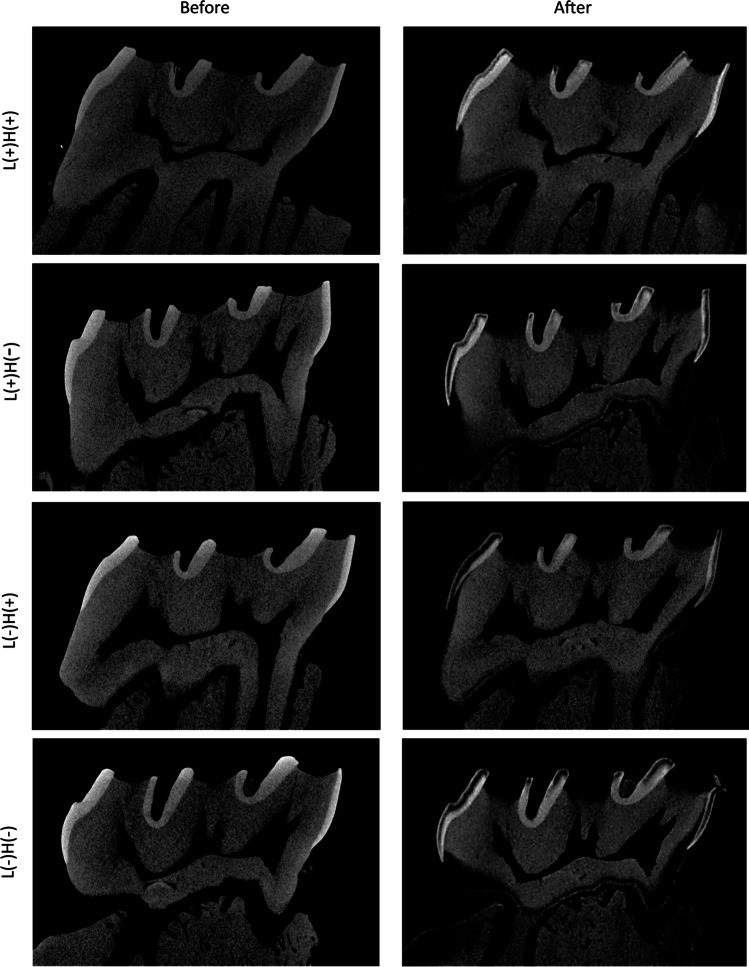
Representative micro-CT images of rat molars obtained before and after treatment, followed by additional 3-day exposure to *Streptococcus mutans* biofilm. A radiolucent layer was observed in both enamel and dentin after treatment, followed by additional exposure to *S. mutans* biofilm, irrespective of the treatment groups

**Fig. 8:**
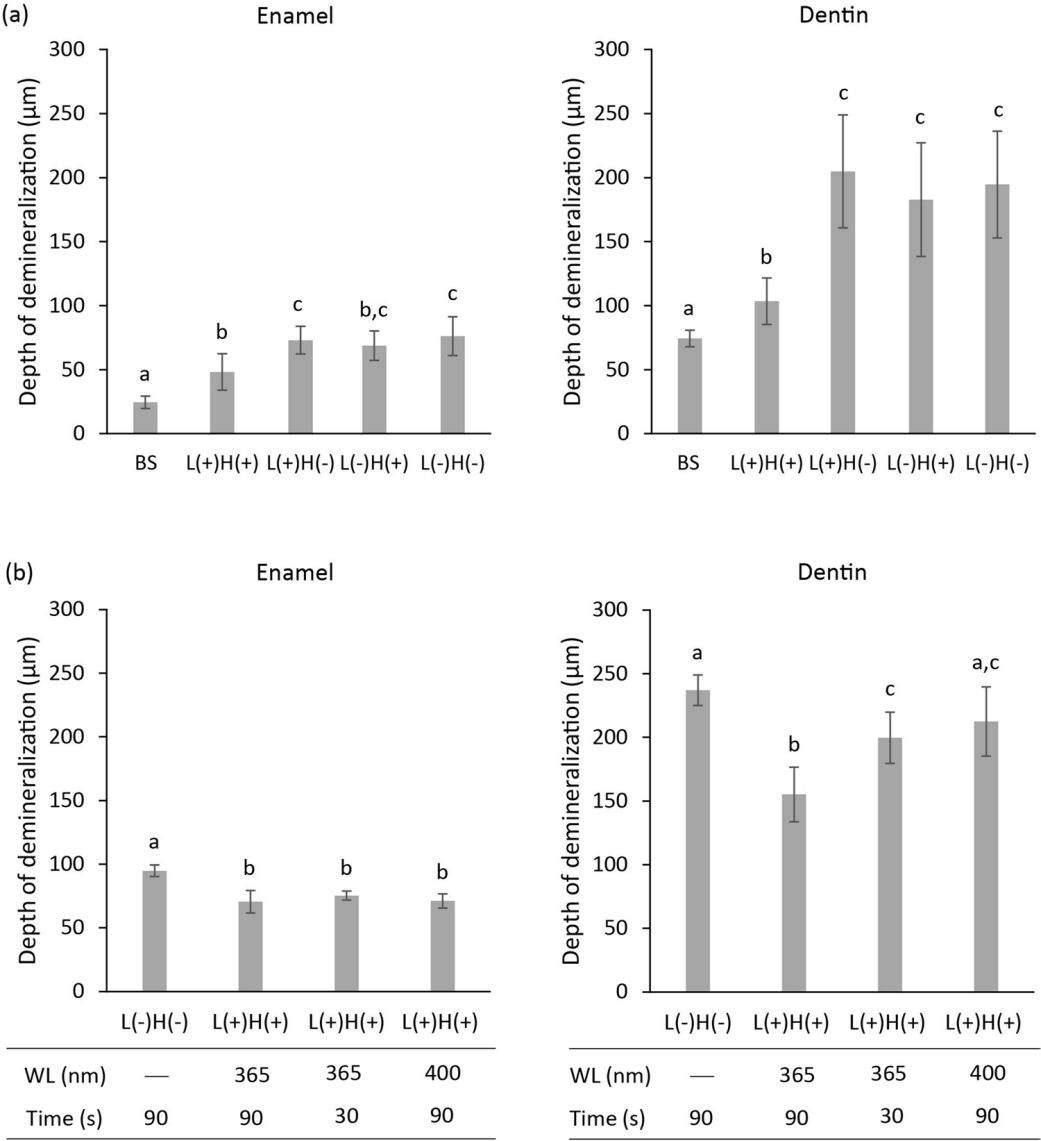
Depth of radiolucent layer in the micro- CT images of enamel and dentin. The initial caries group (baseline: BS) showed the lowest value in both enamel and dentin (**a**). Among the four treatment groups, the L( +)H( +) group showed significantly lower values in both enamel and dentin as compared to the L( +)H( −), L( −)H( +), and L( −)H( −) groups. The influence of irradiation time (30 or 90 s) and light wavelengths (365 or 400 nm) in H_2_O_2_ photolysis treatment on the depth of the radiolucent layer in enamel was limited (**b**). In contrast, the L( +)H( +) treatment with longer irradiation time and shorter wavelength of light showed increased inhibitory effect on demineralization in dentin. The values and error bars indicate the mean and standard deviation, respectively (*n* = 6 for each group). Different letters above the columns indicate significant differences (*p* < 0.05) between the different groups

Radiolucent layer was not observed in the micro-CT images obtained before the formation of *S. mutans* biofilm, irrespective of the treatment groups. In contrast, after the formation of *S. mutans* biofilm in BHI-S for 24 h with or without additional 3 days of incubation, all groups showed a radiolucent layer in both enamel and dentin (Fig. 7). The depth of the radiolucent layer varied according to the group. The initial caries group (baseline group) exposed to *S. mutans* biofilm for 24 h without additional incubation time showed the lowest value. Of the four treatment groups in which the samples were additionally incubated for 3 days after each treatment, the L( +)H( +) group showed significantly lower values in both enamel and dentin compared to the L( +)H( −), L( −)H( +), and L( −)H( −) groups (Fig. 8a).

The effects of irradiation time (30 or 90 s) and wavelengths (365 or 400 nm) on the depth of the radiolucent layer in enamel were limited (Fig. 8b). There were no significant differences observed in the depth between 90 s treatment with L( +)H( +) at 365 nm, 30 s treatment with L( +)H( +) at 365 nm, and 90 s treatment with L( +)H( +) at 400 nm groups, although the L( +)H( +) groups showed significantly lower values compared to L( −)H( −) group with 90 s treatment. On the contrary, L( +)H( +) treatment with longer irradiation time and shorter wavelength of light exerted greater inhibitory effect on demineralization in dentin (Fig. 8b). Thus, 90 s treatment with L( +)H( +) at 365 nm resulted in significantly lower values than the other groups.

## Discussion

The present study aimed to illustrate the effect of H_2_O_2_ photolysis treatment on tooth demineralization caused by cariogenic biofilm. The results demonstrated that H_2_O_2_ photolysis treatment was effective not only in killing biofilm-forming *S. mutans* but also in modifying the acidogenicity of the biofilm, and thus, it could deter the progression of tooth demineralization. Therefore, the hypothesis that H_2_O_2_ photolysis would be effective in inhibition of tooth demineralization was accepted.

In the present study, rat molars were used to evaluate demineralization caused by *S. mutans* biofilm; thus, that the findings of this study can be further verified using in vivo rat dental caries model [33, 34] in future studies. Demineralization was first analyzed using micro-CT and EDS. These analyses demonstrated that tooth demineralization caused by *S. mutans* biofilm was characterized by a radiolucent layer, with reduction in mineral density and reduced percentage of calcium and phosphorus in enamel and dentin, which constitute the primary inorganic content of the tooth (i.e., hydroxyapatite). These changes are typical reactions of tooth demineralization [8]. We also confirmed that progression of demineralization could be quantified by measuring the depth of the radiolucent layer in micro-CT images. Thus, the method used to evaluate the inhibitory effect of H_2_O_2_ photolysis treatment on tooth demineralization was reliable.

Previous studies [15, 26] have confirmed that 90 s treatment with 365 nm LED irradiation of 3% H_2_O_2_ [L( +)H( +)] significantly reduced viable bacterial counts in *S. mutans* biofilms more effectively than LED irradiation alone [L( +)H( −)] or H_2_O_2_ treatment without irradiation [L( −)H( +)]. However, even after the L( +)H( +) treatment, approximately 4 log bacterial cells were still alive in the biofilm. These surviving bacteria regrew after treatment when cultured in a fresh broth, and viable bacterial counts increased to 8.4 log CFU/sample, 30 h after treatment. The viable counts in the L( +)H( +) group were significantly lower than the L( +)H( −), L( −)H( +), and L( −)H( −) groups, although the difference was less than 0.5 log CFU/sample. The lower viable count in the L( +)H( +) group might be related to the lag of bacterial regrowth caused by H_2_O_2_ photolysis treatment, which is known as the post-antibiotic effect [35]. In addition to reduced viable counts, acidogenicity of the biofilms treated with L( +)H( +) was also significantly lower than the other treatment groups. When the re-established biofilm in the L( +)H( +) treatment group was exposed to a fresh broth containing 1% sucrose, the pH level recorded was 6.6, which was higher than the critical pH of enamel (5.5) [36, 37] and dentin (6.0) [38], although these values are controversial [39]. Since the pH of the L( −)H( −) group was 5.3, the difference in pH between the L( +)H( +) and L( −)H( −) groups was 1.3. The difference was larger than that of the viable count (0.5 log CFU/sample), even though both pH and viable counts were measured as logarithmic values. This indicates that H_2_O_2_ photolysis treatment affects the process of glycolysis in the re-established *S. mutans* biofilm, which produces organic acids.

In line with the pH measurement, the samples in the L( +)H( +) group showed significantly lower depth of radiolucent layer in both enamel and dentin than those in the L( +)H( −), L( −)H( +), and L( −)H( −) groups. In contrast, when compared to the initial caries group (baseline), the L( +)H( +) group showed significantly higher depth values. These findings suggest that H_2_O_2_ photolysis treatment may be effective in lowering the demineralization rate caused by *S. mutans* biofilm; however, it could not completely prevent tooth demineralization under the treatment conditions employed in this study. The inhibitory effect of H_2_O_2_ photolysis on tooth demineralization, especially in dentin, was dependent on LED irradiation time and wavelength of light. In dentin, the inhibitory effect was observed in the following order: 90 s treatment with L( +)H( +)at 365 nm > 30 s treatment with L( +)H( +)at 365 nm ≥ 90 s treatment with L( +)H( +) at 400 nm. The longer the irradiation time and the shorter the wavelength, the more hydroxyl radicals are generated in the reaction system of H_2_O_2_ photolysis [11, 12, 32]. Thus, it is reasonable to consider that H_2_O_2_ photolysis treatment with longer irradiation time and shorter wavelength of light would be more effective in killing or inactivating biofilm-forming *S. mutans*, thereby lowering the demineralization rate.

This study had some limitations. First, the *S. mutans* biofilm was continuously exposed to 1% sucrose during the incubation period, due to which the demineralization rate would be much higher as compared to dental caries in the oral cavity. Second, the mode of demineralization may be different from that observed in an in vivo dental caries model in rats. Under the experimental conditions of this study, demineralization occurred at the outer surface of the enamel and the cusp tips where dentin was not covered with enamel. However, dental caries in rats is frequently observed at the bottom of the fissures [33, 34]. Lastly, demineralization was caused by a single species of *S. mutans* to simplify the model, although dental plaque is a multi-species biofilm; thus, the factors affecting the development of dental caries are much more complicated and arduous to replicate. Therefore, the results of the present study should be interpreted taking these limitations into account.

Within the limitations of this study, it was suggested that H_2_O_2_ photolysis not only exerted potent bactericidal effect but also prevented lowering of the pH in regrowth biofilms, the extent of which depended on LED wavelength and irradiation time. Therefore, it can be concluded that H_2_O_2_ photolysis, in particular, irradiation of 3% H_2_O_2_ with 365 nm LED light for 90 s, suppresses tooth demineralization caused by *S. mutans* biofilm. However, it should be noted that 365 nm LED irradiation at 500–2000 mW/cm^2^ may affect the dental pulp and induce tertiary dentin formation, even though the treatment elicited little infiltration of inflammatory cells. [22]. Although there is a possibility to positively utilize tertiary dentin formation in the treatment of dental caries to protect the vital pulp, the irradiation conditions should be optimized before clinical application, in terms of both efficacy and safety.

## Declarations

### Disclaimer

The funders had no role in the study design, data collection, and interpretation, or the decision to submit the work for publication.
